# Exposure Knowledge and Perception of Wireless Communication Technologies

**DOI:** 10.3390/ijerph121114177

**Published:** 2015-11-06

**Authors:** Frederik Freudenstein, Luis M. Correia, Carla Oliveira, Daniel Sebastião, Peter M. Wiedemann

**Affiliations:** 1 Institute for Technology Assessment and Systems Analysis, Karlsruhe Institute of Technology, Berlin 10178, Germany; 2 Instituto Superior Técnico/INOV-INESC, University of Lisbon, Lisbon 1000-029, PortugalE-Mails: luis.correia@inov.pt (L.M.C.); carla.oliveira@inov.pt (C.O.); daniel.sebastiao@inov.pt (D.S.); 3 Faculty of Social Sciences, University of Wollongong, Wollongong 2522, New South Wales, Australia; E-Mail: peter_wiedemann@uow.edu.au

**Keywords:** radio frequency, electromagnetic field, RF EMF, exposure perception, risk perception, risk communication, risk assessment, base stations, mobile phones

## Abstract

The presented survey investigates risk and exposure perceptions of radio frequency electromagnetic fields (RF EMF) associated with base stations, mobile phones and other sources, the key issue being the interaction between both sets of perceptions. The study is based on a cross-sectional design, and conducted with an online sample of 838 citizens from Portugal. The results indicate that respondents’ intuitive exposure perception differs from the actual exposure levels. Furthermore, exposure and risk perceptions are found to be highly correlated. Respondents’ beliefs  about exposure factors, which might influence possible health risks, is appropriate. A regression analysis between exposure characteristics, as predictor variables, and RF EMF risk perception, as the response variable, indicates that people seem to use simple heuristics to form their perceptions. What is bigger, more frequent and longer lasting is seen as riskier. Moreover, the quality of exposure knowledge is not an indicator for amplified EMF risk perception. These findings show that exposure perception is key to future risk communication.

## 1. Introduction

The usage of wireless communication technologies in our day-to-day life has increased dramatically over the years. This tendency is still ongoing, along with the continuous development of new technologies and related applications. The International Telecommunications Union estimates that there will be over 7 billion mobile phone subscriptions worldwide by the end of 2015 [1].

Therefore, the safe use of wireless technologies is a priority of the regulatory agencies which oversee the radio-frequency electromagnetic fields (RF EMF) that are emitted by all wireless technologies. Until now, no adverse health effects have been established [2], however, RF EMF is evaluated as “possibly carcinogenic” by the International Agency for Research on Cancer (IARC) [3] and there are still some open questions regarding health protection [4]. As a consequence, the policies applied to protect public health differ across the word, but the problem is a difficult one. First, “possibly carcinogenic” sounds dramatic, but it is only a weak indication, and it has categorized other everyday encounters similarly (e.g., coffee, see [5]). In the evaluation scheme of IARC, “possibly carcinogenic” is the weakest category that still points towards a carcinogenic effect [6] asking for extra protection and precautionary measures. Paradoxically, such measures, e.g., the prohibition of siting mobile phone base stations in the vicinity of kindergartens and schools, may amplify public concerns [7,8], and lead to less acceptance of wireless technologies in the society. Nevertheless, because wireless technologies are an indispensable part of the ongoing digital revolutions that are shaping the success of national economies, there is a general interest from political authorities in the broad public acceptance of these technologies.

This raises the question about the way of reaching both the goal of public health protection and improved acceptance of wireless technologies. One answer refers to the minimization of exposure, which can be seen as a technical approach acceptance issue. This is the approach taken by LEXNET (low EMF exposure future networks) [9], an ongoing research project supported by the European Commission under the FP7 work programme. It aims to develop effective mechanisms to reduce at least 50% of the public exposure to RF EMF, without compromising the quality of service of wireless communications technologies (see also [10]).

Nevertheless, even a technical approach to the acceptance of wireless communications technologies has to be based on some assumptions about how people perceive and evaluate EMF issues. At the core is the question of whether people will value exposure reduction, *i.e.*, it is a matter of how people’s RF EMF risk perceptions are linked to their RF EMF exposure perceptions.

## 2. Background and Research Questions

In what follows, four research questions are discussed with respect to available evidence and remaining open issues:

- How accurate is the intuitive exposure perception?
- How are people’s risk perceptions related to their intuitive exposure perceptions?
- How accurate is intuitive knowledge about the influence of RF EMF exposure characteristics on health risks?
- Does intuitive knowledge about the influence of RF EMF exposure characteristics on health risks influence people’s RF EMF risk perception?

In the Eurobarometer Study from 2010 [11], people were asked whether several devices and systems were regarded as possible sources of EMF. About 40% of the respondents did not know that base stations and mobile phones emit EMF, and 70% of respondents were unaware of the emissions from wireless computer networks. It seems that people have problems with exposure perception, *i.e.*, identifying exposure sources and their radiation properties. Baliatsas *et al.* [12] also reported a poor correlation between perceived exposure and actual exposure estimates in their research about possible associations between exposure and physical health symptoms.

To our knowledge, there are only a few studies that explore the relationship between RF EMF risk and exposure perceptions. MacGregor *et al.* [13] showed that perceived exposure and perceived health consequences are related, and that a perceived high risk of health effects leads to high exposure perceptions. Another study by Freudenstein *et al.* [14] indicates that exposure plays an important role in shaping risk perception.

There are also only a few studies on people’s knowledge of RF EMF. The study of Cousin and Siegrist [15] shows knowledge gaps in concepts of interaction patterns from mobile phones and base stations, and the related changes in the amount of exposure. People have a low level of knowledge about RF EMF exposure, and they are also unaware that a mobile phone has an antenna and the same basic functionalities as a base station. They perceive distance to the base station as a protecting factor, yet all the while the fact that they themselves hold a transmitting device close to their heads goes unheeded.

The link between subjective knowledge and risk perception is another topic in the RF EMF social science research. There are some findings from our research group with respect to various RF EMF exposure sources [16], which raise doubts as to whether the knowledge of exposure characteristics plays a crucial role in EMF risk perception.

MacGregor’s *et al.* [13] study revealed that a higher knowledge is associated with amplified risk perception. In line with that, Freudenstein *et al.* [17] asked how knowledge of the impact of exposure characteristics—such as number of sources, frequency of exposure, and duration of exposure—on potential health risks are reliable predictors of people’s RF EMF risk perceptions. Interestingly, the distance to the exposure source was not a significant predictor; furthermore, a better knowledge about the impact of exposure characteristics on potential health risks is related to a higher RF EMF risk perception.

However, there are some difficulties in relating exposure characteristics to potential health risks. RF EMF measurements indicate that the distance to a base station is not always a reliable indicator for exposure strength [18]. Other aspects, such as the effect of duration of exposure on potential health risks, are disputed in science. There is some evidence from the Interphone study [19] that heavy users of mobile phones have an elevated risk for brain tumours; however, there is no evidence for a dose-effect-relationship with respect to RF EMF [20].

Of further interest is whether the crucial factors predicting the RF EMF risk perception depend on the exposure source. It could be the case that lay people take different characteristics into account regarding various RF EMF exposure sources, e.g., regarding mobile phones and base stations. Indications, which point in this direction, could be found in a RF EMF perception study for base stations and mobile phones that was based on a sample of citizens from Serbia and Montenegro [16].

## 3. Sample and Methods

### 3.1. Sample

The survey was conducted online in summer 2013, collecting results from 838 Portuguese citizens with a mean age of about 36 years, and a distribution of 58% male and 42% female. The majority of the respondents were well educated, with a mean of 16.1 education years. About 57% live in big cities or suburbs, 31% in a town or a small city, and about 12% in rural areas. Most of the respondents are in paid work (75%).

### 3.2. Method

The online survey [21] consisted of 28 main questions to measure respondents’ intuitive risk and exposure perception. The respondents were asked to assess various exposure sources with respect to their exposure strength. RF EMF sources of different exposure strength as well as a non-EMF radiating device were presented in a randomized order (TV sets, WLAN router, mobile phones or mobile communication masts—*i.e.*, the corresponding base stations) and evaluated on a 5-point Likert scale from 1 (very low intensity) to 5 (very high intensity), which is a technique for the measurement of attitudes.

For a standardized measurement of the RF EMF risk perception of various exposure sources, picture-guided scenarios were used, presenting daily exposure situations of people in an accessible way. The pictures referred to the following exposure situations: mobile communication mast on a school roof, being exposed by WLAN router in close proximity, making mobile phone calls, and watching television. A single question to measure risk perception on a 5-point Likert scale was used (1 = not dangerous, 5 = very dangerous): “How dangerous do you consider this situation to be for (reference to the person on the picture)?”

In addition, respondents were asked to assess the impact of exposure characteristics on the occurrence of potential health risks. In terms of exposure perception, lay people’s subjective knowledge about the impact of exposure features on potential health risks was evaluated, namely, duration, distance, frequency, exposure strength, number of sources, time of day of exposure, and size of source, enabled by the following question: “What do the potential health risks of electromagnetic fields from exposure sources like mobile phones, mobile communication masts, or other devices depend on?” (on a 5-point Likert scale, 1 = Disagree totally, 5 = Agree totally). The questions were: How long are you exposed, how close is the exposure source, how often are you exposed, how strong is the field, how many sources are present, the time of the day during exposure, and the size of the source?

For an in-depth study of possible differences concerning the EMF risk perception in relation to the knowledge base about the impact of exposure characteristics on potential health risk, the subjects were divided into two groups. First, people with better knowledge (n = 117), which scored high (4 or 5 on the 5-point Likert scale) for the following exposure characteristics: duration, strength, distance, frequency and number of sources, and scored low for size and time of the day (1 or 2 on the 5-point Likert scale). The second group, people with inadequate knowledge of exposure characteristics, was operationalized by low scores (≤3 on the 5-point Likert scale) for duration, strength, distance, frequency and number of sources, and high scores (≥3 on the 5-point Likert scale) for size and time of the day (n = 20). Demographic, political, and economic background related items were adapted from the survey platform called “European Social Survey” [22]. All statistical calculations were conducted with IBM SPSS (Statistical Package for the Social Sciences, V20, IBM, New York, NY, USA).

## 4. Results

### 4.1. Real RF EMF Exposure and Exposure Perception

In order to compare RF EMF risk perception with the real user exposure to EMF, the typical radiation levels, expressed in terms of electric field, for the different devices and systems under study are indicated in Table 1. These values are typically observed in normal usage conditions, as reported in Oliveira *et al.* [23] and Kuster [24]. Note that the electric field can be easily measured near to any of the mentioned devices, on the contrary to SAR, whose measurement process is much more complex and difficult to achieve for many scenarios (e.g., people walking in the street submitted to both up- and downlinks exposure from surrounding devices and systems).

**Table 1:** Typical user exposure to various devices (background information. [23,24]).

Device System	User Exposure (Electric Field) [V/m]
Mobile phone	<10
Wireless networks at home	<1
Mobile communication masts	<0.3
TV set	0

Contrary to users’ perception, exposure is usually higher for mobile phones than for mobile communication masts, because mobile phones are operated closer to the user (a few millimeters away or attached to the user), while base stations are usually, at least, 1 to 15 m apart from the user (for micro- and macro-cells, respectively). Exposure to wireless networks at home is generally higher than the one from outdoor base stations, since, again, the former are closer to the user. The TV set is ranked at 0 V/m of exposure, as this device is not a transmitter, rather only receiving the TV signal through its antenna port.

**Figure 1:**
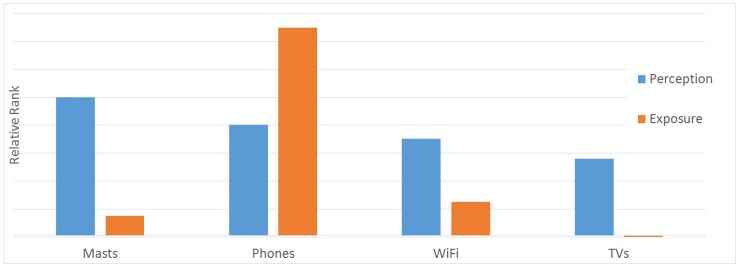
Comparative rank of exposure perception and real exposure.

Table 2 shows the real user exposure compared to the subjective EMF exposure strengths, which the respondents had to estimate (Figure 1), as well as the property of being an RF EMF radiation source, and the tendency for over and underestimation. In Figure 1, the real EMF exposure of the various devices (Table 1) is compared with the subjective exposure perception (Figure 1). Note that, in Figure 1, results are presented as a relative rank, *i.e.*, being normalized to the maximum value for each case (e.g., for exposure perception of base stations, values are normalized to 3.74).

**Table 2:** Ranking means estimated EMF exposure of various devices and systems (Question: “In your opinion, how strong are electromagnetic fields from the following devices or technical systems? “on a 5-point Likert scale from 1 = very low intensity, 5 = very high intensity) and real user exposure in V/m.

Exposure Source	Mean Exposure Perception	Real User Exposure [V/m]	Tendency Over-/Underestimate
Mobile communication masts	3.74	<0.3	↑
Mobile telephones	3.01	<10	↓
Wireless networks at home	2.63	<1	↑
TV set	2.09	0	↑

Note: ↑ = overestimation, **→** = adequate estimation, **↓** = underestimation.

The comparison between real exposure and users’ perceived one clearly shows the tendency for over- and underestimation of exposure for each device/system. This is clearly evident by inspecting Figure 1.

In contrast to lay people’s perspective, the actual user exposure to base stations and mobile terminals is very much different. Compared to the real user exposure, results confirm a clear overestimation of base stations, where exposure is lower (<0.3 V/m) than near-field source mobile phones (<10 V/m). However, note that 10 V/m is a conservative estimation of exposure, but it is still below the recommended threshold values of RF EMF [25].

Concerning wireless networks at home, people think about it as a similar source of exposure to mobile phones, when in fact the real exposure is lower. It is notable that a TV set, which is a non-RF-EMF exposure source, is perceived to emit EMF by lay people. This can be explained by the fact that TV sets are still associated with the old cathode rays tube (CRT) devices, which could emit unintentional x-rays.

### 4.2. Exposure Perception and Risk Perception

**Figure 2:**
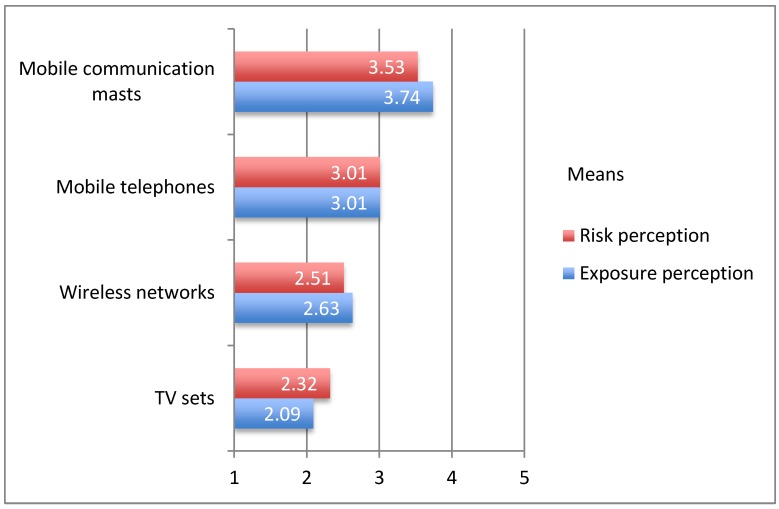
Comparison of means of risk perception and exposure perception of RF EMF devices and systems.

In this section, the link between risk perceptions of possible health effects from different EMF devices and the subjective exposure perception is examined. In terms of the findings regarding the estimated exposure strength, the results indicate that mobile communication masts are perceived as the most intense source. The mean estimated exposure strength is 3.74, on the 5-point Likert scale (1 = very low intensity, 5 = very high intensity), which is followed by lower scores for mobile phones reaching a mean of 3.01, wireless networks at home (mean = 2.63) and TV sets (mean = 2.09). All values are displayed in Figure 2.

The correlations between exposure and risk perceptions for the four RF EMF sources point in the same direction: all correlations are substantial and statistically significant (*p* = 0.000); mobile communication masts, r = 0.450; mobile phones, 0.548; wireless networks, 0.451; TV sets, r = 0.434. The results support the assumption that exposure perception is a good predictor of RF EMF risk perception. Those with high exposure perception tend also to high risk perception.

### 4.3. Subjective Exposure Impact Knowledge and Risk Perception

**Figure 3:**
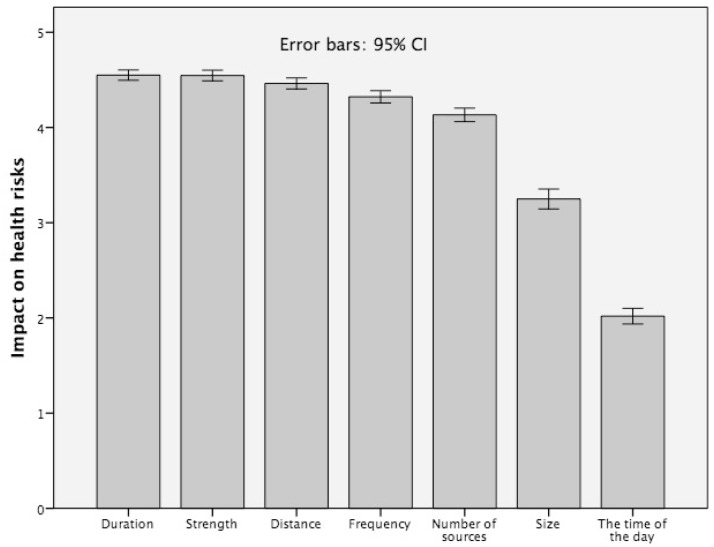
Beliefs about the impact of EMF exposure characteristics on the EMF health risk, 5-point Likert scale 1 = disagree totally to 5 = agree totally (question: “What do the potential health risks of electro-magnetic fields from exposure sources like mobile phones, mobile communication masts, or other devices depend on?”).

An important factor for an adequate assessment of possible adverse effects in exposure situations is the respondents’ subjective knowledge - their beliefs - about the impact of exposure features on potential health risks. As indicated in Figure 3, the exposure characteristics (1) duration of exposure (mean = 4.54); (2) the strength of exposure (mean = 4.54); (3) the distance (mean = 4.46); (4) the frequency of exposure, *i.e.*, how often people are exposed (mean = 4.32); and (5) the number of exposure sources (mean = 4.13) are seen as crucial for health risks. The physical size of the exposure source and the time of the day of exposure, were ranked lower by the respondents (mean = 3.24 and mean = 2.01, respectively). The intervals plotted above the bars in Figure 3 represent the 95% confidence interval, *i.e.*, the interval of values with 95% of probability of containing the population mean.

On a first glance, the results presented in Figure 3 indicate a quite appropriate understanding of how exposure factors may impact potential EMF health risks. They believe that the strength of the exposure source as well as the distance to the source and the duration of the exposure play a crucial role for potential health risks.

Of further interest is how RF EMF risk perception of various exposure sources is actually affected by the respondents’ views regarding the impact of exposure features. Therefore, several regression analyses for different exposure situations were calculated, to investigate the impact of exposure characteristics on EMF risk perception of base stations, mobile phones, WLAN routers and TV sets, as summarized in Table 3, Table 4 and Table 5.

**Table 3:** Linear regression exposure characteristics: dependent variable is risk perception of base stations.

Regression Exposure Characteristics, Base Station	β-Value	*p*
Duration	0.026	0.680
Distance	−0.008	0.874
Frequency	0.105	0.025 *
Strength	−0.060	0.221
Number of sources	0.083	0.056
Time of day	0.089	0.016 *
Size	0.185	0.000 *

Notes: * = significant (level 0.05). R^2^ = 0.118, β represents the relative importance of the predictor variable (various exposure characteristics) in predicting the dependent variable); maximum β is 1. *p* represents the significance level; *p* ≤ 0.05 = sign., *p* ≤ 0.01 = high sign., *p* ≤ 0.001 = highly sign. R^2^ quantifies the explained variance.

The results for base stations demonstrate that the respondents’ risk perception regarding base stations is only slightly affected by factors that are relevant in science. Overall, the amount of the explained variance by the regression model is about 10%.

Most striking is that the belief about the strength of the exposure as a factor influencing potential health risks from EMF exposure is not a significant predictor for risk perception. Frequency seems to play a significant role (β = 0.105, *p* = 0.025) in influencing risk perception of the respondents. This seems not to be an acceptable view, because base stations do not operate in an on- or off-mode like mobile phones.

Furthermore, the size of the base station seems to play the most important role. The time of the day during RF EMF exposure is also a relevant predictor for lay people. At least, the degree of utilization of base stations varies with the time of the day. Taking this dependency into account, the view of “time of the day” as a critical factor is not false. However, another interpretation is possible and this is discussed later.

A slightly different picture is seen for mobile phones: duration (β = 0.112, *p* = 0.027), frequency (β = 0.114, *p* = 0.016) and number of sources (β = 0.088, *p* = 0.045) are significant predictors for EMF risk perception (see Table 4).

**Table 4:** Linear regression exposure characteristics: dependent variable is risk perception of mobile phones.

Regression Exposure Characteristics, Mobile Phones	β-Value	*p*
Duration	0.112	0.027 *
Distance	0.013	0.794
Frequency	0.114	0.016 *
Strength	−0.035	0.474
Number of sources	0.088	0.045 *
Time of day	0.114	0.002 *
Size	0.085	0.030 *

Notes: * = significant (level .05). R^2^ = 0.098. β represents the relative importance of the predictor variable (various exposure characteristics) in predicting the dependent variable; maximum β is 1. *p* represents the significance level; *p* ≤ 0.05 = sign., *p* ≤ 0.01 = high sign., *p* ≤ 0.001 = highly sign. R^2^ quantifies the explained variance.

With the exception of “time of the day” these exposure features may play a significant role for health risks. In addition, again, the belief about the strength of the exposure as a factor influencing potential health risks associated with EMF exposure is not a significant predictor for risk perception. It has to be taken into account that the amount of explained variance by the regression model is low, only amounting to about 10%.

The two remaining sources were also tested in a linear regression model for the independent variables. As seen in Table 5, for a WLAN router, the number of sources and the non-relevant feature time of the day are relevant (β = 0.165 and β = 0.167, both having *p* = 0.000), as can be said for TV sets (β = 0.094, *p* = 0.036 and β = 0.190, *p* = 0.000). Again, the amount of the explained variance is low.

**Table 5:** Linear regressions exposure characteristics: dependent variables are risk perception of WLAN router and risk perception of TV sets.

WLAN Router			TV Sets		
Regression Exposure Characteristics, WLAN	β-Value	*p*	Regression Exposure Characteristics, TV Set	β-Value	*p*
Duration	0.052	0.307	Duration	0.048	0.350
Distance	−0.049	0.324	Distance	−0.073	0.150
Frequency	0.065	0.171	Frequency	0.028	0.565
Strength	−0.040	0.411	Strength	−0.005	0.932
Number of sources	0.165	0.000 *	Number of sources	0.094	0.036 *
Time of day	0.167	0.000 *	Time of day	0.190	0.000 *
Size	0.052	0.186	Size	0.062	0.115

Notes: * = significant (level .05). R^2^ WLAN = 0.100, R^2^ TV set = 0.068. β represents the relative importance of the predictor variable (various exposure characteristics) in predicting the dependent variable; maximum β is 1. *p* represents the significance level; *p* ≤ 0.05 = sign., *p* ≤ 0.01 = high sign., *p* ≤ 0.001 = highly sign. R^2^ quantifies the explained variance.

That exposure characteristics like the time of the day (for all sources) and the size of the source (for base stations and mobile phones) seem to play a significant role in shaping risk perception is notable. In addition, it is striking that the strength of exposure does not play a significant role across all exposure sources. The TV set evaluation indicates a severe problem that lay people have only a restricted knowledge about what is an EMF exposure source and what is not.

### 4.4. Quality of Subjective Exposure Impact Knowledge and Risk Perception

Furthermore, it was examined whether there are differences with respect to risk perceptions between people with *adequate* (n = 20) and *inadequate* (n = 117) knowledge about the impact of exposure characteristics on potential health risks and their subjective exposure perception of various devices.

Table 6 displays the results of a one way analysis of variance with knowledge groups as the independent variable and risk perception as the dependent variable for the four exposure sources: TV set, mobile phones, mobile communication mast (base stations) and wireless networks. There are no statistically significant effects. The quality of knowledge of the effects of exposure characteristics on potential RF EMF risks does not influence EMF risk perceptions.

**Table 6:** One way analysis: knowledge groups as independent variable and EMF risk perception of various exposure sources as dependent variables.

Source of Exposure	Mean Group Adequate Knowledge	Mean Group Inadequate Knowledge	F	*p*	η^2^
TV set	2.12	2.38	1.09	0.297	0.0159
Mobile telephones	2.89	2.80	0.11	0.737	0.0016
Mobile communication masts	3.19	3.43	0.68	0.410	0.0100
Wireless network at home	2.49	2.36	0.26	0.608	0.0038

Notes: (5-point Likert scale from 1 = not dangerous, 5 = very dangerous). Indicated: means of knowledge groups; F-values; p represents the significance level, η^2^ represents the effect size.

These findings raise doubts about whether knowledge of the impact of exposure characteristics on potential health risks plays a crucial role in EMF risk perception. A closer look at other factors that might influence RF EMF risk perceptions is required.

## 5. Strength and Limitations of the Study

A strength of the presented study is the operationalization and measurement of risk and exposure perceptions. It is based on a picture guide approach. Each of the four exposure situations was illustrated by one picture. This approach improved the standardization of the stimulus material and the calibration of the respondents’ answers. A further strength is the restriction to four main research questions that helped to avoid the look-elsewhere-effect, *i.e.*, false positive findings that emerge through multiple testing.

It has to be noted that our sample is not a randomly selected probability sample, where all members of the study population have the same chance of participating in the study. The online survey was conducted by advertising via e-mail, and therefore is only a limited representation of the general public in Portugal. People without Internet access could not be taken into consideration. The age distribution has a wide spectrum (15 to 78 years), but overall the study participants are on average younger than the Portuguese population. The respondents also belong to a well-educated societal group.

In addition, we used a non-experimental approach. Therefore, the regressions and correlations indicate only associations. Strict causal interpretation of the associations is not possible. Nevertheless, the chosen approach, based on a large community sample, allows for testing of relationships between exposure perception, exposure knowledge, and risk perception.

## 6. Discussion

Our findings are in accordance with the psychometric risk perception paradigm [26]. Voluntary risks, such as exposure from mobile phones, are seen as less risky than those that are imposed by other non-voluntary ones, such as exposure from base stations. Usually, risks that are taken voluntarily have some benefits, which are the reason why these risks are taken. An early study conducted by Gregory and Mendelsohn [27] supports this assumption: it revealed that risk ratings are affected by benefit perception. Further research has indicated that the perception of benefits leads to a positive affect, which then reduces perceived risks [28].

Furthermore the study provides insights into what the Portuguese public knows about RF EMF exposure and how they perceive EMF exposure from various sources, and what determines RF EMF risk perceptions.

First the results indicate that risk perception is linked to exposure perception. The presence of this link could be proven across exposure sources, *i.e.*, for base stations, mobile phones and WLAN routers and even for a non-EMF source, like TV sets. The conclusion is: Exposure perception and risk perceptions are related. Those with high exposure perception tend also to high risk perception.

Through comparing perceived exposure with real one some differences become evident. Near-field exposure is underestimated and far-field exposure is overestimated. It indicates that EMF risk perception is determined by people’s subjective beliefs on EMF exposure, and not by objective facts. It replicates findings from our previous study [16].

Additionally, the misinterpretation of TV sets as an exposure source indicates the lack of respondents’ ability to accurately assess exposure sources. This is in line with the results from the Eurobarometer study [11], where various sources of EMF could not be identified by the respondents. Further efforts are necessary to provide information on RF EMF exposure characteristics that can be easily understood.

Regarding the issue of how exposure characteristics do impact potential RF EMF health risks, it seems that people have some intuitive understanding that is based on simple heuristics: What is bigger, more frequent and longer lasting is riskier. Additionally, it is interesting that our respondents used these simple heuristics in a source-specific manner, *i.e.*, different patterns emerged for various RF EMF exposure sources. However, the low level of explained variance in linear regression models point to the fact that people’s RF EMF risk perceptions are only loosely related to their beliefs or subjective knowledge about how exposure characteristics influence potential RF EMF health risks. Our findings demonstrate some distortions and misunderstandings. Most striking is that the strength of exposure does not play any role, but time of the day does. With respect to the latter, we assume that people believe that they are more vulnerable during the night (because they sleep), and therefore RF EMF exposure will have a higher adverse impact on them.

A related issue is that the quality of the beliefs about the impact of exposure characteristics on potential RF EMF related health risks is not related to RF EMF risk perception. Our study provides some evidence that risk perception is not significantly different for the presented two groups of knowledge quality. Of course this has to be replicated in a further study in order to exclude bias and chance. It might be the case that our operationalization of good and poor exposure knowledge is too crude for a meaningful analysis of the relationship between the quality of exposure knowledge and risk perception.

However, if true, it seems to be a result that challenges current thinking. It does not mean that information campaigns that seek to promote exposure reduction are useless. It should be taken into account that the information per se—providing the facts—does not automatically convince doubters or people with strong beliefs, even if these beliefs are wrong. Therefore, further efforts are required in order to analyze the link between quality of knowledge and risk perception in more detail, as well as to develop methods for helping the public to improve its knowledge about RF EMF exposure.

## 7. Conclusions

Overall, the results demonstrate that exposure perception plays a crucial role for RF EMF risk perception. Future risk communication should take into account this dependency. Three major tasks follow from this: (1) supporting people to assess the strength of exposure by comparing various exposure sources; (2) additional research into how beliefs about exposure characteristics are linked to risk perception; and (3) assessing how the acceptability of wireless networks can be influenced by RF EMF exposure reduction.

Better knowledge about what is a RF EMF exposure source and how an exposure source may contribute to the overall exposure of an individual is required. Here comparisons may help. For instance, it should be made clear that usually a cell phone has much higher impact on personal RF EMF exposure than a base station.

Of course, the comparisons of RF EMF exposure sources require some minimal knowledge about how exposure characteristics - for instance the duration of exposure and the distance to the exposure source - interact. Without such knowledge it seems difficult to understand why a cell phone might be more relevant than a base station for personal exposure. Here, more sophisticated research is needed.

The last issue refers to exposure reduction. It should be tested whether the implication of our findings is true, *i.e*., that exposure perception is related to acceptance of wireless technologies via risk perception. In other words, it should be analyzed, how exposure reduction might influence, for instance, the acceptability of a base station in one´s own neighborhood.
